# Death of Dupuytren

**Published:** 1835

**Authors:** 


VII. *Death of Dupuytren.* We copy from the *Boston Medical and Surgical Journal* the following notice of the distinguished Surgeon:

"This very distinguished operator died in Paris on the 8th of February, after a lingering illness of seven months, aged 57. He has left the reputation of being the first operating surgeon in France, and probably in Europe. Domestic affliction preyed upon his mind for the last few years of his life. He has left Madame Beaumont, his only daughter, a fortune of nearly 7,000,000 franks, besides a legacy of 200,000 francs to found a chair of medico-chirurgical pathology. He has also left 100,000 crowns to found an asylum for twelve aged physicians. The annals of surgery do not afford the name of an individual so extensively known; and it is doubtful whether any man has existed who has performed so many operations in surgery, or who has exerted a greater professional influence throughout the civilized world."

